# Sildenafil for treating patients with COVID-19 and perfusion mismatch: a pilot randomized trial

**DOI:** 10.1186/s13054-021-03885-y

**Published:** 2022-01-03

**Authors:** Mario G. Santamarina, Ignacio Beddings, Felipe Martinez Lomakin, Dominique Boisier Riscal, Mónica Gutiérrez Claveria, Jaime Vidal Marambio, Nicole Retamal Báez, Cristian Pavez Novoa, César Reyes Allende, Paulina Ferreira Perey, Miguel Gutiérrez Torres, Camila Villalobos Mazza, Constanza Vergara Sagredo, Sebastian Ahumada Bermejo, Eduardo Labarca Mellado, Elizabeth Barthel Munchmeyer, Solange Marchant Ramos, Mariano Volpacchio, Jorge Vega

**Affiliations:** 1 Radiology Department, Hospital Naval Almirante Nef, Subida Alesandri S/N., 254000 Viña del Mar, Provincia de Valparaíso Chile; 2 Radiology Department, Hospital Dr. Eduardo Pereira, Valparaiso, Chile; 3 Radiology Department, Hospital Clínico San Borja Arriarán, Santiago, Chile; 4 Intensive Care Unit, Hospital Naval Almirante Nef, Viña del Mar, Chile; 5 Escuela de Medicina, Facultad de Medicina, Universidad Andres Bello, Viña del Mar, Chile; 6 Respiratory Department, Hospital Naval Almirante Nef, Viña del Mar, Chile; 7 General Internal Medicine Department, Hospital Naval Almirante Nef, Viña del Mar, Chile; 8 Infectious Disease Department, Hospital Naval Almirante Nef, Viña del Mar, Chile; 9 Hospital Pharmacy Department, Hospital Naval Almirante Nef, Viña del Mar, Chile; 10 Radiology Department, Centro de Diagnóstico Dr. Enrique Rossi, Buenos Aires, Argentina; 11 Departamento de Medicina, Escuela de Medicina, Universidad de Valparaíso, Viña del Mar, Chile

**Keywords:** COVID-19, Sildenafil, Subtraction CT angiography, Ventilation–perfusion ratio, Blood gas analysis, Mechanical ventilation, Intensive care unit, Length of stay

## Abstract

**Background:**

SARS-CoV-2 seems to affect the regulation of pulmonary perfusion. Hypoperfusion in areas of well-aerated lung parenchyma results in a ventilation–perfusion mismatch that can be characterized using subtraction computed tomography angiography (sCTA). This study aims to evaluate the efficacy of oral sildenafil in treating COVID-19 inpatients showing perfusion abnormalities in sCTA.

**Methods:**

Triple-blinded, randomized, placebo-controlled trial was conducted in Chile in a tertiary-care hospital able to provide on-site sCTA scans and ventilatory support when needed between August 2020 and March 2021. In total, 82 eligible adults were admitted to the ED with RT-PCR-confirmed or highly probable SARS-COV-2 infection and sCTA performed within 24 h of admission showing perfusion abnormalities in areas of well-aerated lung parenchyma; 42 were excluded and 40 participants were enrolled and randomized (1:1 ratio) once hospitalized. The active intervention group received sildenafil (25 mg orally three times a day for seven days), and the control group received identical placebo capsules in the same way. Primary outcomes were differences in oxygenation parameters measured daily during follow-up (PaO_2_/FiO_2_ ratio and A-a gradient). Secondary outcomes included admission to the ICU, requirement of non-invasive ventilation, invasive mechanical ventilation (IMV), and mortality rates. Analysis was performed on an intention-to-treat basis.

**Results:**

Totally, 40 participants were enrolled (20 in the placebo group and 20 in the sildenafil group); 33 [82.5%] were male; and median age was 57 [IQR 41–68] years. No significant differences in mean PaO_2_/FiO_2_ ratios and A-a gradients were found between groups (repeated-measures ANOVA *p* = 0.67 and *p* = 0.69). IMV was required in 4 patients who received placebo and none in the sildenafil arm (logrank *p* = 0.04). Patients in the sildenafil arm showed a significantly shorter median length of hospital stay than the placebo group (9 IQR 7–12 days vs. 12 IQR 9–21 days, *p* = 0.04).

**Conclusions:**

No statistically significant differences were found in the oxygenation parameters. Sildenafil treatment could have a potential therapeutic role regarding the need for IMV in COVID-19 patients with specific perfusion patterns in sCTA. A large-scale study is needed to confirm these results.

*Trial Registration*: Sildenafil for treating patients with COVID-19 and perfusion mismatch: a pilot randomized trial, NCT04489446, Registered 28 July 2020, https://clinicaltrials.gov/ct2/show/NCT04489446.

**Supplementary Information:**

The online version contains supplementary material available at 10.1186/s13054-021-03885-y.

## Background

To date, more than 247 million infections and more than 5 million deaths due to SARS-CoV-2 have been reported worldwide [1]. Although vaccines have been developed in record time, and already more than 6893 million vaccine doses have been administered worldwide, there is still a long time until global immunity [1]. The longer the virus continues to spread unchecked, the higher the risk of more deadly or contagious variants emerging.

Most patients infected with the SARS-CoV-2 virus develop a self-limiting infection and present with mild symptoms or appear asymptomatic. Nevertheless, up to 20% of patients may develop a rapid progression to acute hypoxemic respiratory failure, leading to ventilation support, admission to the intensive care unit (ICU), and eventually death [2]. In addition, severe gas-exchange impairment can occur in even the early stages, with only minor lung airspace disease appearing in CT images [2].

SARS-CoV-2 seems to affect the regulation of pulmonary perfusion, resulting in early oxygenation impairment and hypoxemia in the course of the disease [3, 4]. The binding of the virus with its angiotensin-converting enzyme 2 (ACE2) receptor causes a renin–angiotensin system imbalance with excessive activation of the angiotensin-converting enzyme/angiotensin II/angiotensin type 1 receptor pathway, which is in line with a reduction in the angiotensin-converting enzyme 2/angiotensin-(1–7)/MAS receptor pathway [5]. Elevated levels of endothelial angiotensin II also stimulate the generation of reactive oxygen species, which are responsible for nitric oxide (NO) breakdown [6]. Local NO depletion leads to vasoconstriction, which establishes a progressive V/Q mismatch with extensive areas of well-aerated lung parenchyma in CT images but hypoperfused in subtraction CT angiography (sCTA) images, that function as alveolar dead space [7].

These findings suggest that evaluation of therapies oriented to improve pulmonary perfusion in the early phases of mild to moderate COVID-19 pneumonia should be considered. Sildenafil could be a good alternative because it is a widely used orally administrable drug, is cheaper, more stable, and easier to handle than inhaled NO (iNO) [8]. Thus, this trial was designed as a pilot study to evaluate the potential role of sildenafil in improving oxygenation among hospitalized patients and to assess its possible impact in ICU admission, requirement of invasive ventilatory support, and survival among patients with COVID-19 pneumonia.

## Methods

Ethical approval was obtained from IRB of Hospital Naval Almirante Nef and Universidad Andres Bello, Chile (register number: 022/2020). All participants provided their written informed consent. The protocol was developed according to the CONSORT guidelines. This trial is registered on ClinicalTrials.gov (NCT04489446).

### Design and setting

This triple-blinded, randomized, placebo-controlled trial occurred at Hospital Naval Almirante Nef, Viña del Mar, Chile. Patients, physicians, researchers, and laboratory analysts were unaware of treatment allocations. All data were handled anonymously by the statistical analyst using coded information to preserve patient privacy. A single non-blinded physician (JVS) monitored patient safety daily. The results were concealed from other researchers using sealed opaque envelopes.

### Participants, recruitment strategy, and randomization

From August 2020 to March 2021, 82 adult patients were admitted to the emergency department (ED) with highly probable SARS-COV-2 infection that was later confirmed with RT-PCR. After meeting clinical criteria for hospitalization, patients underwent an admission CT angiogram with sCTA during the first 24 h of admission, which showed perfusion abnormalities in sCTA, and afterward asked if they would like to participate in the trial.

They also had moderate CT severity scores and severe sCTA perfusion scores, with iodine maps showing a predominance of hypoperfused healthy parenchyma over hyperperfused areas of airspace disease. Two validated radiologists (MS and IB) assessed sCTA perfusion images at admission. Borderline cases were decided by consensus. The sCTA protocol and analysis of CT severity score and sCTA perfusion score are presented in Additional file 1. Patients were enrolled once hospitalized and were excluded if at admission they required invasive mechanical ventilation (IMV), had an indication of limitation of therapeutic efforts, or presented global respiratory failure with hypercapnia; pulmonary hypertension; chronic use of phosphodiesterase 5 inhibitors; or contraindications for sildenafil. Additional file 2: Table S1 presents all eligibility criteria. Of these individuals, 40 were eligible.

To achieve descriptive estimators of sildenafil’s efficacy with acceptable precision in 95% confidence intervals, we proposed randomizing 40 patients (20 per group) for comparisons. An independent statistician randomized participants using permuted blocks with a 1:1 ratio. The *ralloc* package in Stata v16.0® (StataCorp LP, 1996–2020) was used to compute allocation sequences.

### Interventions

The active intervention group received 25 mg of generic sildenafil orally three times daily (every 8 h) for 7 days. The control group received identical placebo capsules. The remaining treatment was decided by the attending physician. All patients were followed up daily for 2 weeks.

### Outcome measures

Primary endpoints were changes in oxygenation parameters [(PaO_2_/FiO_2_ ratio and alveolar–arterial oxygen gradient (A-a gradient)] calculated before, one hour, and eight hours after the first intervention, and daily for 7 days. Arterial lines were installed in all patients for measurements. The procedure for estimation of oxygenation parameters is shown in Additional file 1. Secondary endpoints were ICU admission, non-invasive mechanical ventilation, IMV, and in-hospital mortality. Adverse reactions from sildenafil or the placebo were considered crucial biosafety endpoints.

### Participant characteristics

Patient information included sex, age, relevant comorbidities (Charlson comorbidity index), laboratory tests upon admission (SARS-CoV-2 RT-PCR, blood count, creatinine, C-reactive protein, lactate, fibrinogen, ferritin, creatine kinase, lactate dehydrogenase, ultra-sensitive troponin, and D-dimer), and imaging results.

### Statistical analysis

Basic descriptive statistics (means, medians, proportions, interquartile ranges (IQR), etc.) were examined. Fisher’s exact test evaluated univariate associations of categorical variables. Quantitative variables were compared using the Mann–Whitney or Student’s *t*-tests according to data distributions and variances. 95% confidence intervals were constructed whenever appropriate.

A two-way repeated-measures analysis of variance (ANOVA) assessed sildenafil’s effects. Sequential laboratory measurements were the within-group variance variables, and treatment allocation was the between-group variance term. Sphericity was assessed using the epsilon statistic. The sphericity assumption was considered violated with epsilon values less than 0.75. Greenhouse–Geisser correction was used on *p*-values to minimize type-1 error rates associated with noncompliance with this assumption.

Secondary endpoints including ICU admission, initiation of IMV, and mortality rates were evaluated with Kaplan–Meier survival analysis and compared using log-rank statistics. Due to the pilot study’s nature, no prespecified subgroup analyses were planned. All analyses were performed using Stata v16.0® (StataCorp LP, 1996-2020) under the intention-to-treat principle by a blinded statistician.

## Results

Trial enrolment was carried out in two periods: between August 20, 2020, and October 19, 2020, and between December 14, 2020, and March 31, 2021. There was an enrolment pause between October 20, 2020, and December 13, 2020, due to imaging software limitations. Enrolment resumed once the software limitation was completely resolved.

**Fig. 1:**
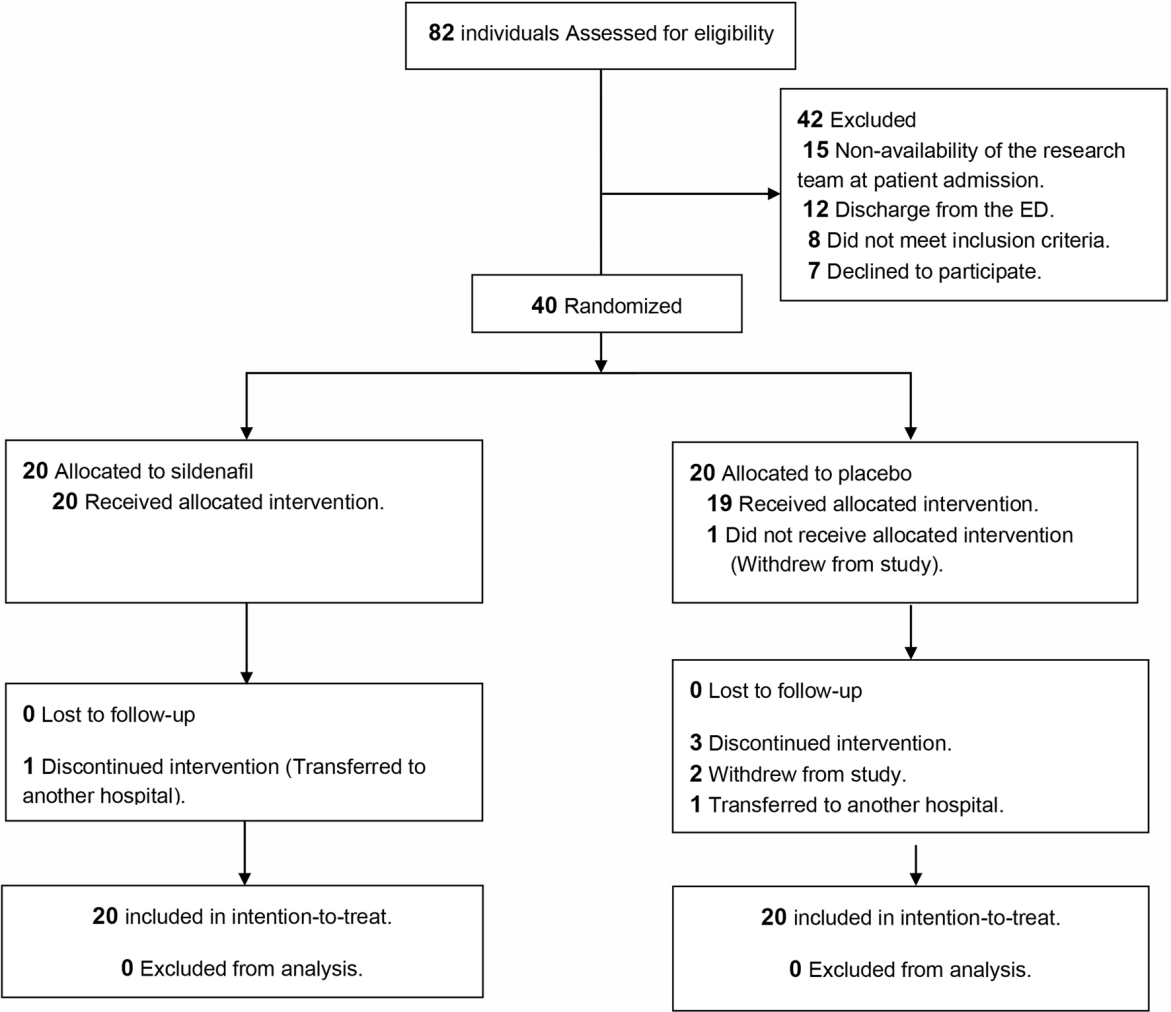
Trial profile. Flow of participants in a pilot study of sildenafil versus placebo for treating patients with COVID-19 with specific perfusion patterns in sCTA

Out of 82 eligible patients, 42 were excluded (Fig. 1). The reasons for exclusion were non-availability of the research team at patient admission, discharge from the ED, the existence of exclusion criteria and the patient's decision not to participate. A total of 40 participants were randomized to receive either sildenafil or placebo (20 per group) using a computer-generated algorithm and sealed envelopes. There were 35 patients (87.5%) who completed their follow-up, and two (5%) were transferred to other hospitals which were also followed up and included in the analysis. Three patients (7.5%) opted to discontinue the study drug, and all of them had been allocated to receive the placebo. A detailed description of the study participants is provided in Table 1.

**Table 1:** Baseline characteristics

Characteristic	Sildenafil group (*n* = 20)	Placebo group (*n* = 20)	Total (*n* = 40)
Age (years) (median, IQR)	59 (41–69)	57 (45–67)	57 (41–68)
Male sex (*n*, %)	16 (80%)	17 (85%)	33 (82.5%)
Symptom duration (days) (median, IQR)	9 (7–11)	8 (6.5–10)	8 (7–10)
Active smoker (*n*, %)	3 (15%)	2 (10%)	5 (12.5%)
Asthma (*n*, %)	1 (5.0%)	1 (5.0%)	2 (5.0%)
Chronic obstructive pulmonary disease (*n*, %)	0 (0%)	0 (0%)	0 (0%)
Arterial hypertension (*n*, %)	10 (50%)	10 (50%)	20 (50%)
Diabetes mellitus (*n*, %)	4 (20%)	4 (20%)	8 (20%)
Charlson comorbidity index score (median, IQR)	1 (0–3)	1 (0–3)	1 (0–3)
Ambient oxygen (*n*, %)	6 (30%)	7 (35%)	13 (32.5%)
Awake prone positioning (*n*, %)	5 (25%)	6 (30%)	11 (27.5%)
High-flow nasal cannula (*n*, %)	1 (5%)	1 (5%)	2 (5%)
Oxygen support flow (Lts/min, median, IQR)	1 (0–4.5)	2.5 (0–5)	2 (0–5)
*Laboratory*			
Arterial pH (median, IQR)	7.45 (7.43–7.48)	7.45 (7.43–7.47)	7.45 (7.44–7.47)
Arterial oxygen partial pressure (PO_2_, mmHg) (median, IQR)	83.3 (70.7–87.5)	84.1 (76.5–95.0)	83.3 (75.4–90.1)
Arterial carbon dioxide partial pressure (mmHg) (median, IQR)	33 (31.2–34.7)	36.2 (31.3–37.7)	33.4 (31.2–36.9)
Bicarbonate (mEq/Lt) (median, IQR)	23.7 (21.3–25.1)	24.2 (22.6–26.9)	24.0 (22.4–25.6)
PaO_2_/FiO_2_ ratio (median, IQR)	308 (216–366)	299 (220–359)	302 (216–365)
A-a gradients (median, IQR)	51.6 (40.3–133)	71.0 (37.3–149)	66 (40–146)
D-Dimer (ng/mL) (median, IQR)	271 (191–404)	287 (170–545)	284 (190–424)
Absolute lymphocyte count (cells/mm^3^) (median, IQR)	860 (730–1200)	860 (680–1120)	860 (680–1160)
Lactate dehydrogenase (UI/L) (median, IQR)	320 (252–355)	310 (271–488)	316 (254–398)
C-Reactive protein (mg/L) (median, IQR)	90.3 (35.6–187.0)	98.0 (63.3–139.0)	92.5 (56.6–179)
Serum creatinine (mg/dL) (median, IQR)	0.87 (0.79–1.0)	0.78 (0.73–0.87)	0.82 (0.75–0.93)
Serum blood urea nitrogen (mg/dL) (median, IQR)	18.2 (14.9–20.6)	15.2 (11.0–18.1)	16.7 (12.5–19.6)
*Computed tomography*			
Pulmonary embolism (*n*, %)	0 (0%)	1 (5.0%)	1 (2.5%)
Vascular beaded appearance (*n*, %)	11 (55%)	10 (50%)	21 (52.5%)
CT severity score (median, IQR)	9 (7–10)	9 (7.5–10)	9 (7–10)
sCTA perfusion score (median, IQR)	10 (8–10)	10 (8–10)	10 (8–10)

The demographic characteristics were well balanced between groups. Most patients were male (33 patients, 82.5%), and the median age was 57 (IQR 41–68) years. The median symptom duration was 8 days (IQR 7–10 days), and 13 patients (32.5%) were breathing room air at randomization. The overall comorbidity burden was low with a median Charlson Comorbidity Index of 1 point (IQR 0–3 points).

The most common diseases were arterial hypertension (20 patients, 50%) and diabetes mellitus (8 patients, 20%). There were 33 patients (82.5%) who received intravenous dexamethasone as part of their care, while 30 patients (80%) received ceftriaxone, and 4 patients received azithromycin (20%). Empirical therapeutic anticoagulation was started in 5 (12.5%) patients. No significant differences were seen regarding coadjuvant therapies between study groups (Table 2).

**Table 2:** Coadjuvant therapy

Treatment	Sildenafil group (*n* = 20)	Placebo group (*n* = 20)	Total (*n* = 40)	
Dexamethasone (*n*, %)	17 (86%)	16 (80%)	33 (82.5%)	
Azithromycin (*n*, %)	4 (20%)	4 (20%)	8 (20%)	
Ceftriaxone (*n*, %)	16 (80%)	14 (70%)	30 (75%)	
Therapeutic anticoagulation (*n*, %)	2 (10%)	3 (15%)	5 (12.5%)	

**Fig. 2:**
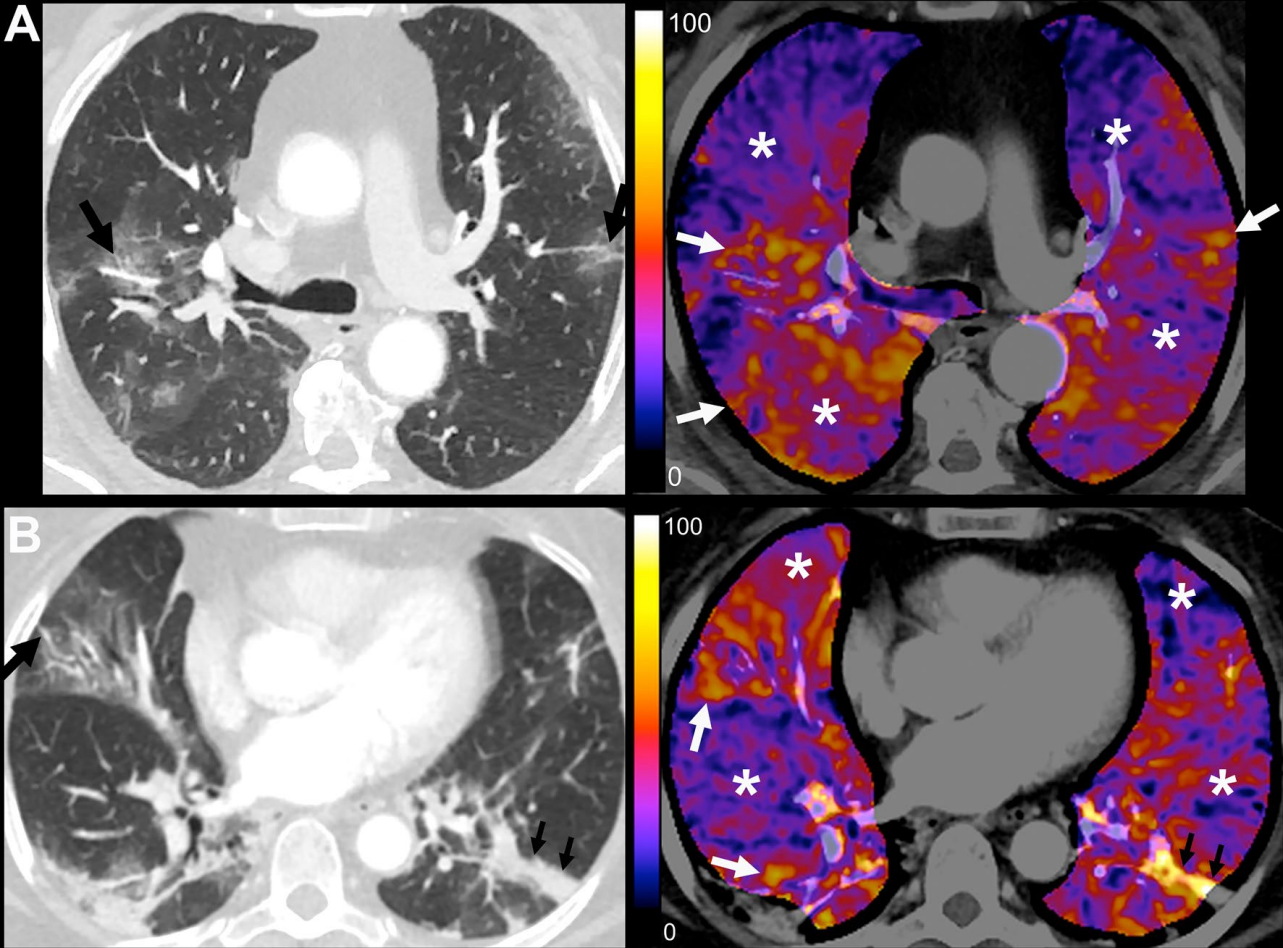
Conventional and color map sCTA images within 24 h of admission to the hospital and before administration of the first dose of the allocated intervention. **A** Placebo Arm. 70-year-old male patient before receiving the first dose of placebo with RT-PCR-confirmed COVID-19 at 6 days since symptom onset. Admission Charlson score was 3, A-a gradient was 75.6, and PaO_2_/FiO_2_ ratio was 322. D-dimer was 272 ng/mL. He was admitted to the ICU, managed with IMV, and died almost 7 weeks after admission. CT severity score: 9; sCTA perfusion score: 10. Moderate lung involvement with patchy ground-glass opacities in both lungs with vascular dilatation in small peripheral subsegmental pulmonary arterial branches, some of them with a varicose appearance (black arrows). Severe hypoperfusion abnormalities in apparently normal lung parenchyma (*). Some areas of ground-glass opacities show marked hyperperfusion, most likely due to vasoplegia (white arrows). **B** Sildenafil Arm. 64-year-old female patient before receiving the first dose of sildenafil with RT-PCR-confirmed COVID-19 at 7 days since symptom onset. Admission Charlson score was 2, A-a gradient was 109.9, and PaO_2_/FiO_2_ ratio was 207. D-dimer was 308 ng/mL. She was admitted to the ICU, managed with high flow nasal cannula, and stayed in the hospital for 14 days until discharge. CT severity score: 9; sCTA perfusion score: 10. Moderate lung involvement with patchy ground-glass opacities in both lungs and laminar atelectasis (small black arrows). There is vascular dilatation in small peripheral subsegmental pulmonary arterial branches (black arrow). Severe hypoperfusion abnormalities in apparently normal lung parenchyma (*). Some areas of ground-glass opacities show marked hyperperfusion, most likely due to vasoplegia (white arrows). Linear atelectasis shows increased perfusion in lower left lobe (small black arrows)

Patients showed only mild hypoxemia at baseline. The median PaO_2_/FiO_2_ ratio was 302 (IQR 216–365), and the median A-a gradient was 66 (IQR 40–146) mmHg. Patients in the sildenafil arm tended to have higher PaO_2_/FiO_2_ and lower partial pressures of CO_2_ than the placebo arm, but not significantly. The median CT severity score was 9 (IQR 7–10) points, and the overall median sCTA perfusion score was 10 (IQR 8–10) points, indicating moderate airspace compromise due to COVID-19 and severe perfusion alterations (Fig. 2). Vascular beaded appearance was common, with 21 (52.5%) patients showing this finding in CT angiography. Evidence of pulmonary embolism was found in 1 patient (2.5%).

**Fig. 3:**
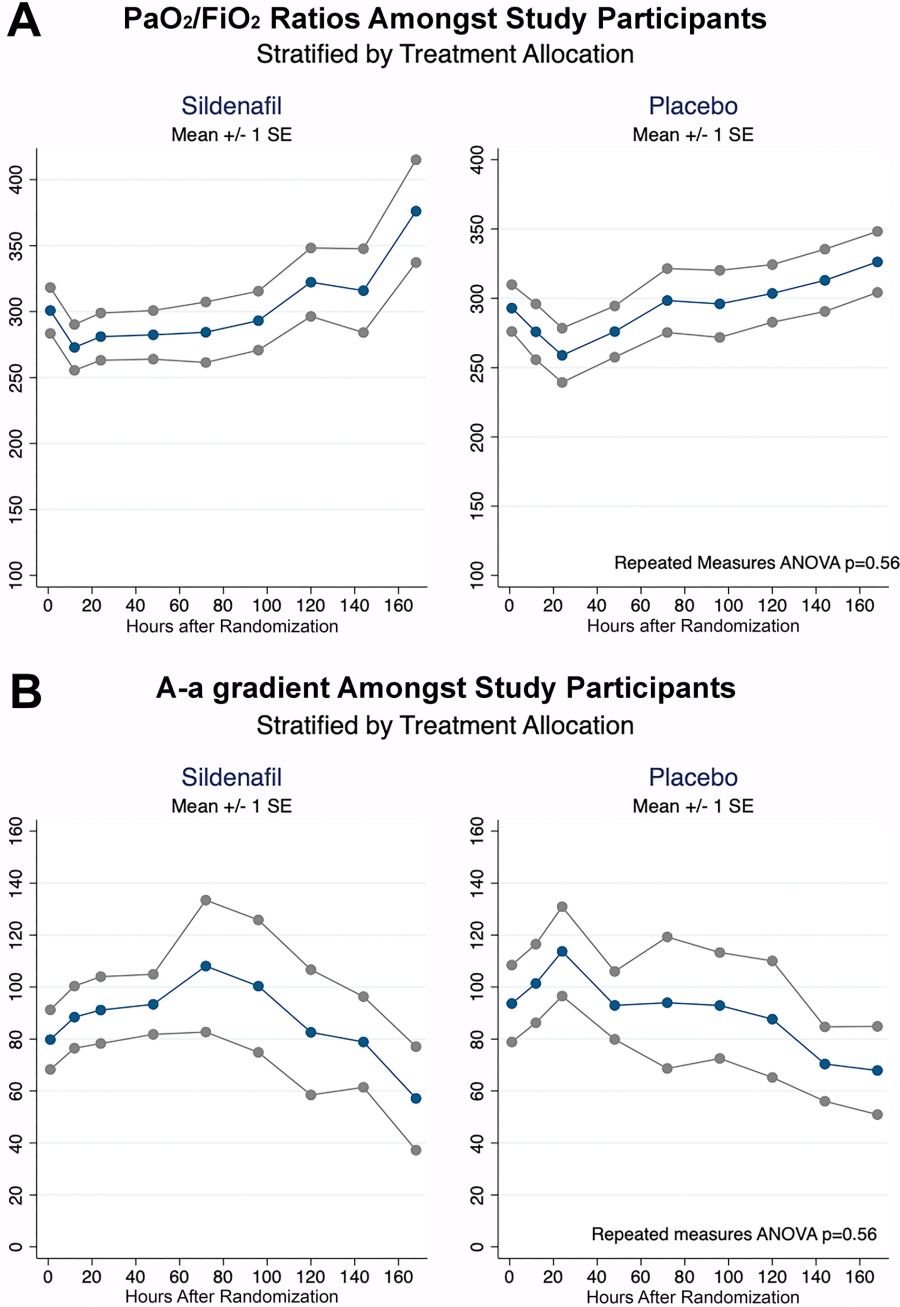
Primary outcomes. **A** Observed PaO_2_/FiO_2_ ratios among study participants. Stratified by treatment allocation. **B** Observed A-a gradient among study participants. Stratified by treatment allocation

The mean PaO_2_/FiO_2_ ratios at one hour after treatment administration tended to be higher among those in the sildenafil group, but not significantly (274 IQR 230–338 vs. 267 IQR 216–310 *p* = 0.56). On the other hand, the mean A-a gradients tended to be lower among patients receiving sildenafil (81.6 IQR 44–124 vs. 104 IQR 44–149 *p* = 0.56). When trends over time were analyzed, no differences in mean PaO_2_/FiO_2_ ratios were observed between study groups (repeated-measures ANOVA *p* = 0.67). A similar finding was seen in A-a gradients between study groups (repeated-measures ANOVA *p* = 0.69). Figure 3 shows the trends of alveolar gas analysis between study groups.

Twenty-two patients were randomized during the first period of our study and 18 during the second. A 1:1 ratio of patients receiving sildenafil or placebo was maintained in both periods of recruitment. Patients admitted during the second phase tended to show higher perfusion CT scores (median 10, IQR 10–10 points vs. 8 IQR 8–10 points, *p* < 0.001) than those who entered our trial during the first phase. No significant differences were seen regarding the extension of pulmonary compromise (*p* = 0.37). The aforementioned alterations in perfusion patterns also resulted in lower median Pa/Fi ratios (median 231 IQR 204–322 vs. 350 IQR 273–372, *p* < 0.01) and higher alveolo-arterial gradients (median 127 IQR 55.4–158 vs. 45 IQR 27.6–1206, *p* < 0.02) during the second phase of our study.

Eight patients (20%) were admitted to the ICU during their follow-up, 3 (15%) being in the sildenafil arm and 5 (25%) in the placebo group. These differences were not statistically significant (HR 0.68, 95% CI 0.16–2.87, *p* = 0.59). Respiratory support using a high-flow nasal cannula was started in 6 patients, which were evenly distributed between the placebo and sildenafil arms (*p* = 0.95).

**Fig. 4:**
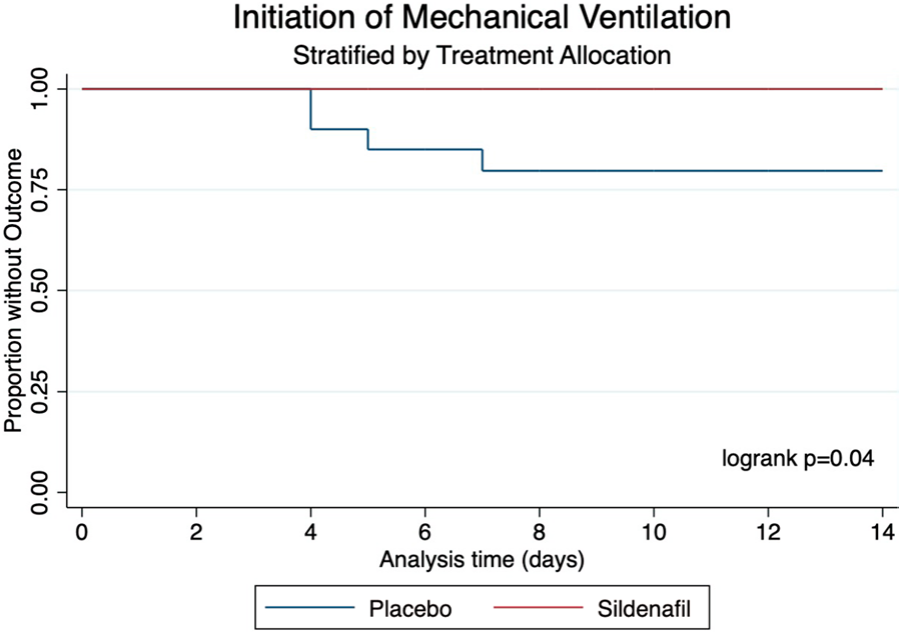
Initiation of invasive mechanical ventilation. Stratified by treatment allocation

Mechanical ventilation had to be initiated due to severe respiratory failure in 4 (10%) patients, and all of them were in the placebo arm. When survival analyses were conducted, a statistically significant difference was found between study groups (logrank *p* = 0.04), as shown in Fig. 4. The median duration of mechanical ventilation was 21 (IQR 8.5–36.5) days.

Patients in the sildenafil arm also showed a significantly shorter median length of stay in the hospital than the placebo group (9 IQR 7–12 days vs. 12 IQR 9–21 days, *p* = 0.04). The median duration of stay in the ICU was 15 (IQR 7–42) days among patients receiving a placebo and seven days (IQR 7–8) in those given sildenafil (*p* = 0.46). Only one patient died during follow-up and was in the placebo arm. A detailed description of these outcomes is provided in Table 3.

**Table 3:** Study outcomes

Outcome	Sildenafil group (*n* = 20)	Placebo group (*n* = 20)	Total (*n* = 40)	*P* value
*Clinical outcomes*				
In-hospital mortality (*n*, %)	0 (0%)	1 (5.0%)	1 (2.5%)	> 0.99^a^
Intensive care unit (ICU) admission (*n*, %)	3 (15%)	5 (25%)	8 (20%)	0.59^a^
Initiation of high-flow nasal cannula (*n*, %)	3 (15%)	3 (15%)	6 (15%)	0.95^a^
Initiation of invasive mechanical ventilation (*n*, %)	0 (0%)	4 (20%)	4 (10%)	0.04^a^
Median duration of mechanical ventilation (days) (IQR)	–	21 (8.5–36.5)	–	–
Median ICU stay (days) (IQR)	7 (7–8)	15 (7–42)	7.5 (7–28.5)	0.46^b^
Median hospital stay (days) (IQR)	9 (7–12)	12 (9–21)	11 (8–14)	0.04^c^
*Laboratory outcomes*				
Mean PaO_2_/FiO_2_ ratio one hour after treatment administration (SD)	274 (230–338)	267 (216–310)	275 (230–314)	0.56^c^
Mean A-a gradient 1 h after treatment administration (SD)	81.6 (44–124)	104 (44–149)	91.5 (32.7–175)	0.56^c^
*Adverse events*				
Headache (*n*, %)	2 (10%)	5 (25%)	7 (17.5%)	0.41^d^
Dizziness (*n*, %)	4 (20%)	2 (10%)	6 (15%)	0.66^d^
Blurred vision (*n*, %)	0 (0%)	0 (0%)	0 (0%)	–
Flushing (*n*, %)	2 (10%)	0 (0%)	2 (5%)	0.49^d^
Nausea (*n*, %)	0 (0%)	4 (20%)	4 (10%)	0.11^d^
Nasal congestion (*n*, %)	3 (15%)	2 (10%)	5 (12.5%)	> 0.99^d^
Other (*n*, %)	1 (5%)	0 (0%)	1 (2.5%)	> 0.99^d^

^a^Logrank statistic

^**b**^Mann–Whitney rank sum test

^c^Student's *T* test

^d^Fisher's exact test

SD, standard deviation; IQR, interquartile range

Adverse events were common among the included participants**;** most of these were mild and tended to be more frequent among patients in the placebo group. The most common were headache (7 patients, 17.5%), dizziness (6 patients, 15%), and nasal congestion (5 patients, 12.5%). Patients receiving sildenafil tended to report dizziness, flushing, and nasal congestion more frequently than those receiving the placebo. No severe adverse events attributable to sildenafil were found in this study (Table 3).

## Discussion

The mean PaO_2_/FiO_2_ ratio and mean A-a gradient one hour after treatment administration showed no differences between both groups. However, there were statistically significant differences in the clinically relevant outcomes of median hospital stay and, most importantly, initiation of IMV, which favored the sildenafil group. There is a 4% probability that the reductions in hospitalization length and initiation of IMV were random results.

The mechanisms of progression of pulmonary involvement due to COVID-19 pneumonia are not well understood. Severe gas-exchange impairment can occur in even the early stages, with only minor lung airspace disease in CT images.

When the virus reaches the airspace, it produces local inflammation and edema, and consequently airspace disease that is evidenced in CT images.

Nevertheless, there are different points of view regarding the pathophysiology of COVID-19-ARDS [3, 4, 12–17]. It is very likely that the prevalent pathophysiological mechanism is initiated on the vascular side of the pulmonary unit [18]. Once the virus infects the endothelial cell, it probably depletes ACE2, while inflammatory signaling molecules are released and inflammatory cells are recruited [16]. This leads to a release of vasoactive agents, with marked activation of the endothelium that leads to increased vascular permeability and vascular smooth muscle relaxation, resulting in a loss of hypoxic vasoconstriction that could explain the vasodilation and vascular beaded appearance observed in areas of airspace disease in early COVID-19 [12, 16, 19]. Later, significant local endothelial dysfunction could lead to potential activation of thrombotic and fibrinolytic pathways, and eventually angiogenesis [21–23].

Then, the virus spreads to the pulmonary blood circulation, and the binding of SARS-CoV-2 to ACE2 from endothelial cells in more distant well-ventilated areas produces an imbalance of the renin–angiotensin system and endothelial dysfunction [20].

This leads to decreased NO production, resulting in vasoconstriction and hypoperfusion, establishing a progressive V/Q mismatch that manifests as extensive areas of apparently healthy but hypoperfused lung that functions as alveolar dead space [7, 24, 25]. This pilot study was therefore proposed to assess a potential therapeutic role of sildenafil in compensating the vascular tone imbalance with predominant vasoconstriction in areas of apparently healthy lung parenchyma, as well as to prevent the lungs from becoming more inflamed with excessive respiratory drive.

Sildenafil inhibits phosphodiesterase enzyme type 5 (PDE5), which normally breaks down cyclic guanosine monophosphate (cGMP). In pulmonary circulation, NO is mainly synthesized by endothelial NO synthase (eNOS), which diffuses into vascular smooth muscle cells, where it stimulates cyclic guanosine monophosphate (cGMP) production from soluble guanylyl cyclase [26]. cGMP produces vasodilatation in the pulmonary vasculature, among other actions [26]. Inhibition of PDE5 by sildenafil has the potential to augment NO-related vasodilatation in regions of perfusion demand [9]. Furthermore, it has anti-inflammatory and antiaggregating effects [10, 11]. Sildenafil could also be a good alternative to iNO because it is a widely used oral drug and is cheaper, stable, and easier to handle than iNO [8].

Despite being a pilot study, there were no statistically significant differences in the baseline characteristics of both groups, which provided a solid basis for comparison. There were statistically significant differences in observed reductions in median hospital stay and, most importantly, initiation of IMV, which favored the sildenafil group. These findings are highly relevant for patients, physicians, and administrators since they could translate into some relief for the currently overburdened healthcare systems around the globe. However, due to the dissociation between laboratory results and clinical outcomes, and the pilot nature of the study, these findings should be interpreted with caution, and no causality can be assumed. Several factors should be kept under consideration. First, due to the low sample size, this pilot study lacks power to demonstrate statistically significant differences in blood gas analyses. Second, laboratory parameters (PaO_2_/FiO_2_ ratio and A-a gradient) present several limitations and may not reliably reflect the patient's condition [27, 28]. Third, other potential effects of NO on endothelial function could be playing a role in improving clinical outcomes. These effects might not have been measurable with the primary endpoints that were established a priori during the design of this trial. Fourth, the renin–angiotensin system imbalance and inflammatory process produce endothelial dysfunction with eNOS uncoupling, causing a decrease in NO production and consequently a decrease in cGMP [29–31]. cGMP also activates PDE5, which in turn inactivates cGMP [32]. The lower availability of NO observed in the initial stages of mild-to-moderately ill COVID-19 patients [33] could potentially translate to lower initial availability of cGMP. It is possible that this lower initial availability of cGMP could make it more difficult to detect the effects of a PDE5 inhibitor such as sildenafil in early stages of treatment. This could have led to the non-statistically significant differences in PaO_2_/FiO_2_ ratio and alveolar–arterial gradient seen in the early days of hospitalization. It could be possible to hypothesize that belatedly, the neutralization of the virus by the immune response [34] and the reduction in endothelial dysfunction could progressively increase the availability of cGMP by means of both increased production of NO [32] by endothelial cells and decreased inactivation by PDE5 with the use of sildenafil, progressively helping to compensate for the vascular tone imbalance, which could explain the relative tendency toward improved oxygenation parameters in the latest measurements in Fig. 3. A larger study is necessary to demonstrate whether these different tendencies between groups persist.

The main limitation of this study is that it had a relatively low sample size given its pilot nature. However, it must be kept in mind that this study was not designed to find statistically significant differences in clinical or laboratory outcomes, but to achieve descriptive estimators that allow for sample size calculation in future studies using sildenafil as part of COVID-19 treatment. To date, there has been no previous information regarding the potential effects of sildenafil in COVID-19. Another limitation is the fact that for patients to be included in this study, the sCTA had to show areas of decreased perfusion in the well-aerated lung, which is usual in patients with COVID-19 pneumonia with mild-to-moderate airspace disease [7]. However, sCTA is a new postprocessing technique, it has not been as extensively validated as dual-energy computed tomography (DECT), and both techniques are not so widely available [7, 23, 35]. Finally, there is another sCTA perfusion pattern in COVID-19 patients that is usually found in more severely ill patients, with more extensive lung air space compromise, which usually show characteristics of respiratory distress. This pattern shows diffusely increased blood perfusion toward areas of consolidated as well as aerated lung parenchyma, and it is consistent with findings observed in other studies [36–39]. sCTA perfusion maps allowed us to exclude patients with this particular perfusion pattern from the study, as the authors consider that the systemic effect of sildenafil could be detrimental in this subset of patients, potentially favoring blood flow steal toward vasoplegic and hyperperfused areas of non-aerated (consolidated) lung parenchyma.

## Conclusions

In conclusion, no statistically significant differences were found in the oxygenation parameters. Nevertheless, differences were found between groups in hospitalization length and initiation of IMV. This pilot study shows that there could be a potential therapeutic role for sildenafil in a subset of patients with specific perfusion patterns in sCTA and provides descriptive estimators for sample size calculation in future larger trials.

## Supplementary Information

**Additional file 1**.** I- Supplementary Methods**. 1- Subtraction CT Angiography Protocol. 2- Image analysis: CT Severity Score. 3- Image analysis: sCTA Perfusion Score. 4- Estimation of oxygenation parameters.** II- Supplementary References**.

**Additional file 2: Table S1**. Eligibility criteria

**Additional file 3: Figure S1**. Conventional and color map sCTA axial images. Normal perfusion. Excluded from randomization. 26-year-old female patient. 3 days since symptoms onset, with fever, headache, and cough. PCR was negative for COVID-19. Outpatient management. CT severity score: 0; sCTA perfusion score: 0. Axial CT image with lung windows shows normal lungs. Corresponding color map sCTA image from the same examination shows a normal distribution with a mild smooth gravitational gradient favoring the posterior lungs and no focal perfusion abnormalities.

**Additional file 4: Figure S2**. Conventional and color map sCTA coronal images. Predominance of hyperperfused areas of airspace disease over hypoperfused healthy parenchyma. Excluded from randomization. 73-year-old female patient. RT-PCR confirmed COVID-19, 7 days since symptom onset. PaO2/FiO2 ratio was 133. She was admitted to the ICU, managed with IMV. She was in the hospital 21 days until discharge. CT severity score: 13; sCTA perfusion score: 10. Extensive lung air space disease, in which there is diffusely increased blood perfusion toward areas with airspace opacification. There is an extension predominance of hyperperfused areas (black asterisks) of airspace disease over hypoperfused (white asterisks) in apparently healthy parenchyma.

**Additional file 5: Table S2**. Estimated effective fractional concentration of oxygen in inspired gas of low-flow and reservoir oxygen delivery devices.

## Data Availability

The datasets used and/or analyzed during the current study are available from corresponding author on reasonable request.

## Abbreviations

A-a gradient: Alveolar–arterial oxygen gradient
ACE2: Angiotensin-converting enzyme 2
cGMP: Cyclic guanosine monophosphate
ED: Emergency department
eNOS: Endothelial NO synthase
DECT: Dual-energy computed tomography
ICU: Intensive care unit
IMV: Invasive mechanical ventilation
iNO: Inhaled NO
NO: Nitric oxide
PDE5: Phosphodiesterase enzyme type 5
sCTA: Subtraction computed tomography angiography
