# Supplementary material for: Sildenafil for treating patients with COVID-19 and perfusion mismatch: a pilot randomized trial
Source: Crit Care. 2022 Jan 3;26:1. doi: 10.1186/s13054-021-03885-y (PMC8721481; doi:10.1186/s13054-021-03885-y)
Supplement: Supplementary file 1 — Additional file 1. I- Supplementary Methods. 1- Subtraction CT Angiography Protocol. 2- Image analysis: CT Severity Score. 3- Image analysis: sCTA Perfusion Score. 4- Estimation of oxygenation parameters. II- Supplementary References. [file 13054_2021_3885_MOESM1_ESM.docx]

**ADDITIONAL FILE 1**

**Sildenafil for Treating Patients with COVID-19 and Perfusion Mismatch: A Pilot Randomized Trial**

**Authors:**

Mario G. Santamarina^1,2^ MD; Ignacio Beddings^3^ MD; Felipe Martinez Lomakin^4,5^ MD, MSc; Dominique Boisier Riscal^4^ MD; Mónica Gutiérrez Claveria^6^ MD; Jaime Vidal Marambio^4^ MD; Nicole Retamal Báez^4^ RN; Cristian Pávez Novoa^4^ MD; Cesar Reyes Allende^4^ MD; Paulina Ferreira Perey^7^ MD; Miguel Gutiérrez Torres^7^ MD; Camila Villalobos Mazza^8^ MD; Constanza Vergara Sagredo^4^ MD; Sebastian Ahumada Bermejo^6^ MD; Eduardo Labarca Mellado^4^ MD; Elizabeth Barthel Munchmeyer^8^ MD ; Solange Marchant Ramos^9^ CHEM; Mariano Volpacchio^10^ MD; Jorge Vega^7,11^ MD

^1^ Radiology Department, Hospital Naval Almirante Nef, Viña del Mar, Chile.

^2^ Radiology Department, Hospital Dr. Eduardo Pereira, Valparaiso, Chile.

^3^ Radiology Department, Hospital San Borja Arriaran, Santiago, Chile.

^4^ Intensive Care Unit, Hospital Naval Almirante Nef, Viña del Mar, Chile.

^5^ Universidad Andres Bello, Viña del Mar, Escuela de Medicina, Facultad de Medicina Viña del Mar, Valparaiso, Chile

^6^ Respiratory Department, Hospital Naval Almirante Nef, Viña del Mar, Chile.

^7^ General Internal Medicine Department, Hospital Naval Almirante Nef, Viña del Mar, Chile.

^8^ Infectious Disease Department, Hospital Naval Almirante Nef, Viña del Mar, Chile.

^9^ Hospital Pharmacy Department, Hospital Naval Almirante Nef, Viña del Mar, Chile.

^10^ Radiology Department, Centro de Diagnóstico Dr. Enrique Rossi, Buenos Aires, Argentina.

^11^ Departamento de Medicina, Escuela de Medicina, Universidad de Valparaíso, Chile

**Corresponding Author:** Mario G. Santamarina

Radiology Department, Hospital Naval Almirante Nef, Viña del Mar, Chile.

Subida Alesandri S/N., Viña del Mar, Provincia de Valparaíso, Chile. Zip Code: 254000.

Email address: mgsantama@yahoo.com

**Table of Contents**

**I- Additional file Methods**……..**….……………………………………………….……………...3**

1- Subtraction CT Angiography Protocol……………………………………………………………**3**

2- Image analysis: CT Severity Score……………………………..………………………………..**3**

3- Image analysis: sCTA Perfusion Score………………………………………………………….**4**

4- Estimation of oxygenation parameters…………………………………………………………..**5**

**II- Additional file References…………………………………………………………….............7**

**I- Additional file Methods**

**1- Subtraction CT Angiography Protocol**

Adult patients with RT-PCR-confirmed or high clinical suspicion of SARS-COV-2 infection who satisfied hospitalization criteria underwent a subtraction CT angiography (sCTA) upon admission to the hospital. Imaging data were acquired with multidetector CT (Canon Aquilion Prime 80). Firstly, an unenhanced scan was obtained after placing the patient in a supine position, followed by IV injection of 100 mL iodinated contrast medium at a rate of 5 mL/seg (Visipaque 320, GE Healthcare, Milwaukee, WI). After bolus triggering at the level of the pulmonary artery with a relative threshold of 150 HU, an early pulmonary arterial angiographic phase was obtained, followed by a delayed pulmonary arterial phase eight seconds later. All the exams were performed with the same acquisition parameters of kV (100 kV), automatic exposure control (standard), similar range and field-of-view (FOV: L or LL), collimation (0.5 × 80), pitch factor (1.4), and rotation speed (0.35 s). The acquired images had the same reconstruction parameters of slice thickness (1.0 mm), reconstruction interval (0.8 mm), and convolution filter (mediastinum). The average dose DLP (mGy/cm) was 1063 (range: 701–1946). Breathing instructions were the same for all examinations.

The sCTA technique uses software-based motion correction between unenhanced and enhanced CT scans to obtain an iodine distribution map of the lung parenchyma. Iodine distribution maps of the early and delayed arterial phases were obtained using SureSubtraction software (version 7.0; Canon Medical Systems, Japan). Iodine maps were generated with gray-scale and identical color window tables ranging from blue (low iodine enhancement) to yellow (high iodine enhancement).

**2- Image analysis: CT Severity Score**

Areas of injured parenchyma in both lungs were assessed for a predominant pattern. These were characterized as ground glass opacities, consolidation, and mixed patterns. Airspace compromise was assessed for each of the 5 lobes while considering the extent of anatomic involvement as follows: 0 points for no involvement; 1 point for < 30% involvement; 2 points for 31–60% involvement; and 3 points for > 61% involvement. The resulting CT severity score was the sum of each individual lobar score (range: 0 - 15), which allowed us to classify the extent of pulmonary involvement as mild (1-5), moderate (6-10), or severe (11-15). Iodine distribution maps were evaluated together with the conventional CT images.

**3- Image analysis: sCTA Perfusion Score**

Areas of apparently healthy lung parenchyma in conventional CT images were assessed qualitatively to determine the presence and extent of hypoperfusion, also while considering the normally expected anteroposterior and apicobasal perfusion gradient. Hypoperfusion in iodine distribution maps was graded using the following scoring system (sCTA perfusion score). First, both lungs were divided into five lobes (upper right and left lobes, middle lobe, and lower right and left lobes). Then, the extension of hypoperfusion in apparently healthy lung parenchyma in each lobe was categorized as “normal perfusion” (0 points), “less than 50% of the lobe affected” (1 point), and “50% of the lobe or more affected” (2 points). This resulted in an overall score that ranged from 0 to 10 points, with higher scores indicating more severe hypoperfusion. Using this score, patients were divided into three groups. Patients with 3 points or less were considered to have mild perfusion abnormalities, those with 4–6 points had moderate abnormalities, and those with 7 or more points had severe abnormalities.

Generally, in patients with COVID-19 pneumonia, the areas of non-aerated lung (with ground glass opacities, consolidation, or both) show an increase of pulmonary flow in iodine maps, as well as loss of compensatory hypoxic pulmonary vasoconstriction. Therefore, in patients with more severe airspace compromise in conventional CT images and a higher severity CT score, we find a greater extension of this hyperperfusion in areas of airspace disease in iodine maps. These findings are consistent with other studies in ICU hospitalized patients with severe COVID where they have shown significant lung vasodilation^1-4^.

To meet the inclusion criteria, patients had to have a moderate CT severity score and a severe sCTA perfusion score. In other words, iodine maps had to show an extension predominance of hypoperfused healthy parenchyma over hyperperfused areas of airspace disease. Additional file 3: Fig. S1 shows an example of normal perfusion in healthy parenchyma, and Additional file 4: Fig. S2 shows a predominance of hyperperfused areas of airspace disease over hypoperfused healthy parenchyma, which is an example that does not meet inclusion criteria.

**4- Estimation of oxygenation parameters**

We defined PaO_2_/FiO_2_ ratio and A–a gradient as monitoring parameters in an attempt to determine differences in oxygenation measured daily during follow-up.
Although the ratio of the partial pressure of oxygen in arterial blood (PaO_2_) to the inspired oxygen fraction (FiO_2_) (PaO_2_/FiO_2_ ratio) presents several limitations^5-7^, given its simplicity we decided to use it to quantify the degree of abnormalities in pulmonary gas exchange. To calculate the PaO_2_/FiO_2_ ratio, we divided the arterial pO_2_ from the arterial blood gases, by the FiO_2_ – the fraction (percent) of inspired oxygen that the patient is receiving expressed as a decimal (for example: 30% oxygen = FiO_2_ of 0.30). Although it is difficult to accurately estimate FiO_2_, we considered room air as a fraction of inspired oxygen of 0.21, and in Additional file 5: Table S1 we show the equivalences that we determined from different devices^8-10^.

The A–a gradient helps to assess the integrity of the alveolar-capillary unit and originates due to both physiological right-to-left shunting and a physiological V/Q mismatch caused by gravity-dependent differences in perfusion to various zones of the lungs. The difference between partial pressures of oxygen in alveolar space (partial pressure of oxygen in the alveolar space calculated from the alveolar gas equation) and PaO_2_ is the equation of the A–a gradient.

We calculated the A–a gradient by^11,12^:

A-­a gradient = [FiO_2_ (PA –PH_2_O) – (PaCO_2_/RQ)] – PaO_2_

Where:

FiO_2_ is the fraction of oxygen, PA is atmospheric pressure, and PH_2_O is partial pressure of water vapor. Thus, we carry out the test at sea level, so the PA is 760 mm Hg, and assuming 100% humidity in the alveoli then water vapor pressure at 37°C is approximately 47 mm Hg. We did not adjust alveolar PH_2_O as a function of temperature.

Although PaCO_2_ is partial pressure of carbon dioxide in alveolar space, we replaced it by partial pressure of carbon dioxide in arterial blood as CO_2_ considering that it diffuses efficiently, and RQ is the respiratory quotient (the ratio of CO_2_ production to O_2_ consumption). To standardize, we considered a RQ of 0.8. We did not adjust the A-a gradient as a function of age.

Replacing values ​​that are constant, we estimated the A–a gradient by:

A-­a gradient = [FiO_2_ (760 – 47) – (PaCO_2_/0.8)] – PaO_2_

This calculation distinguishes between pure hypoventilation as an explanation for hypoxemia (in which case, A-­a gradient is normal) and the presence of other mechanisms such as low ventilation-perfusion ratios and right-to- left shunt (in which case, A-­a gradient is increased) ^12^.

**II- Additional file References**

1- Brito-Azevedo A, Pinto EC, de Cata Preta Corrêa GA, Bouskela E. SARS-CoV-2 infection causes pulmonary shunt by vasodilatation. *J Med Virol*. 2021 Jan;93(1):573-575. doi: 10.1002/jmv.26342.

2- Reynolds AS, Lee AG, Renz J, DeSantis K, Liang J, Powell CA, et al. Pulmonary Vascular Dilatation Detected by Automated Transcranial Doppler in COVID-19 Pneumonia. *Am J Respir Crit Care Med*. 2020;202(7):1037-1039. doi:10.1164/rccm.202006-2219LE.

3- Poor HD, Rurak K, Howell D, Lee AG, Colicino E, Reynolds AS, et al. Cardiac index is associated with oxygenation in COVID-19 acute respiratory distress syndrome. *Pulm Circ*. 2021;11(2):20458940211019626. doi:10.1177/20458940211019626

4- Caravita S, Baratto C, Di Marco F, Calabrese A, Balestrieri G, Russo F, et al. Haemodynamic characteristics of COVID-19 patients with acute respiratory distress syndrome requiring mechanical ventilation. An invasive assessment using right heart catheterization. *Eur J Heart Fail*. 2020 Dec;22(12):2228-2237. doi: 10.1002/ejhf.2058.

5- Aboab J, Louis B, Jonson B, Brochard L. Relation between PaO2/FIO2 ratio and FIO2: a mathematical description. Intensive Care Med. 2006 Oct;32(10):1494-7. doi: 10.1007/s00134-006-0337-9.

6- Gattinoni L, Vassalli F, Romitti F. Benefits and risks of the P/F approach. Intensive Care Med. 2018 Dec;44(12):2245-2247. doi: 10.1007/s00134-018-5413-4.

7- Karbing DS, Kjaergaard S, Smith BW, et al. Variation in the PaO_2_/FiO_2_ ratio with FiO_2_: mathematical and experimental description, and clinical relevance. Crit Care. 2007;11(6):R118. doi: 10.1186/cc6174.

8- Hardavella G, Karampinis I, Frille A, Sreter K, Rousalova I. Oxygen devices and delivery systems. *Breathe (Sheff)*. 2019;15(3):e108-e116. doi:10.1183/20734735.0204-2019

9- Fuentes S, Chowdhury YS. Fraction of Inspired Oxygen. [Updated 2021 Jan 17]. In: StatPearls [Internet]. Treasure Island (FL): *StatPearls Publishing*; 2021 Jan-. Available from: <https://www.ncbi.nlm.nih.gov/books/NBK560867/>. Accessed date 2021 Sep 19.

10- Jia S, Hyzy R. Noninvasive Support of Oxygenation. In: Broaddus V, Ernst J, King T, et al. *Murray & Nadel's Textbook of Respiratory Medicine*. 7^th^ edition. Philadelphia, PA, USA: Elsevier Health Sciences; 2022:Chapter 137.
11- Foderaro A, Misra A. Respiratory Failure. In: Wing E, Schiffman F. *Cecil essentials of Medicine*. 10^th^ edition. Philadelphia, PA, USA: Elsevier Health Sciences; 2022:Chapter 20.
12- Bhakta N, Kaminsky D. Pulmonary Function Testing: physiologic and Technical principles. In: Broaddus V, Ernst J, King T, et al. *Murray & Nadel's Textbook of Respiratory Medicine*. 7^th^ edition. Philadelphia, PA, USA: Elsevier Health Sciences; 2022:Chapter 31.
