# Supplementary material for: Sildenafil for treating patients with COVID-19 and perfusion mismatch: a pilot randomized trial
Source: Crit Care. 2022 Jan 3;26:1. doi: 10.1186/s13054-021-03885-y (PMC8721481; doi:10.1186/s13054-021-03885-y)
Supplement: Supplementary file 2 — Additional file 2: Table S1. Eligibility criteria [file 13054_2021_3885_MOESM2_ESM.docx]

**Additional file 2: Table S1. Eligibility criteria**

**Inclusion Criteria:**

1. Adult participant (>18 years old) with high clinical suspicion of a SARS-CoV2 infection at admission to the hospital and later confirmed with RT-PCR.
2. Hypoperfusion in a subtraction computed tomography angiography in healthy lung areas in CT images within 24 hours of admission to the hospital.

**Exclusion Criteria:**

1. Requirement of therapy with nitrates or nitrites.
2. Arterial hypotension at presentation.
3. Recent diagnosis of coronary artery disease (<6 months).
4. Acute heart failure at presentation.
5. Recent stroke (< 6 months).
6. Chronic respiratory failure with CO2 retention.
7. Known hypersensitivity to sildenafil.
8. Advanced liver disease (Child-Pugh class B or higher).
9. Users of cytochrome P450 3A4 inhibitors.
10. Pulmonary hypertension.
11. Chronic users of phosphodiesterase 5 inhibitors.
12. Requirement of invasive mechanical ventilation at admission to the hospital.
13. Decision to limit therapeutic efforts at admission to the hospital.
14. Pregnancy or lactation.
15. History of retinitis pigmentosa.
16. Known obstruction to left-ventricular outflow tract.
17. Chronic kidney disease (stage IV or V).
18. Unwillingness to participate in the trial.
