# Supplementary figures and images for: Sildenafil for treating patients with COVID-19 and perfusion mismatch: a pilot randomized trial
Source: Crit Care. 2022 Jan 3;26:1. doi: 10.1186/s13054-021-03885-y (PMC8721481; doi:10.1186/s13054-021-03885-y)

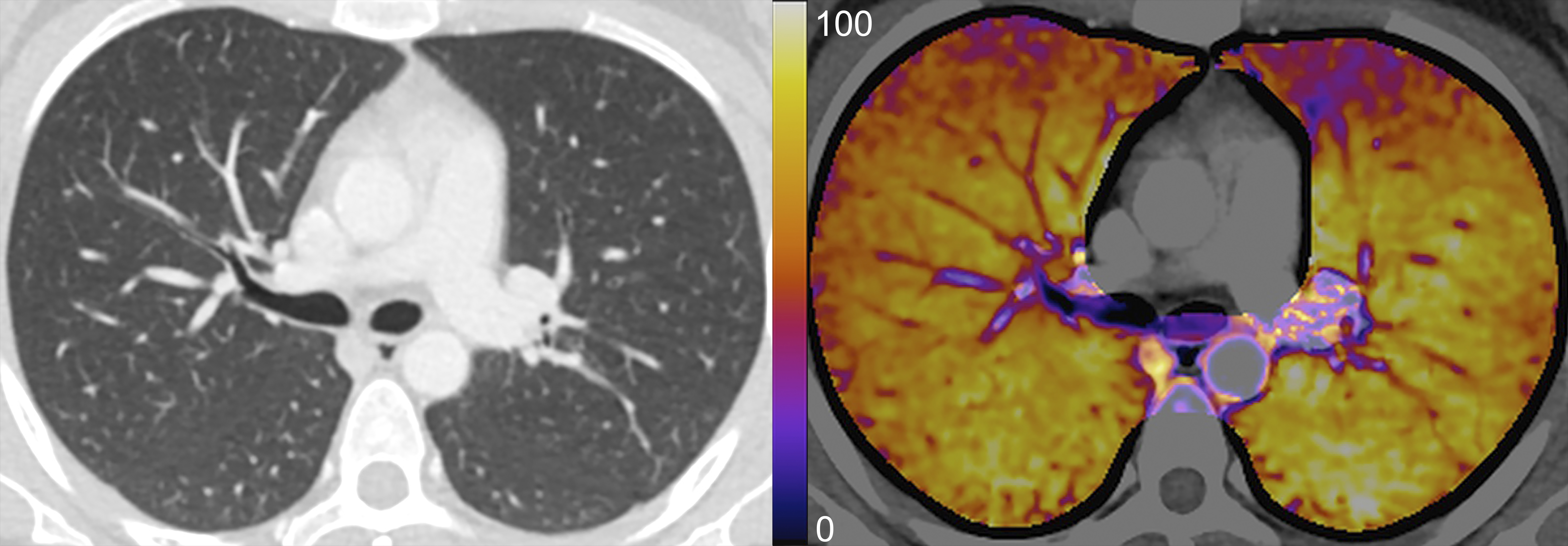

Supplement: Supplementary file 3 — Additional file 3: Figure S1. Conventional and color map sCTA axial images. Normal perfusion. Excluded from randomization. 26-year-old female patient. 3 days since symptoms onset, with fever, headache, and cough. PCR was negative for COVID-19. Outpatient management. CT severity score: 0; sCTA perfusion score: 0. Axial CT image with lung windows shows normal lungs. Corresponding color map sCTA image from the same examination shows a normal distribution with a mild smooth gravitational gradient favoring the posterior lungs and no focal perfusion abnormalities. [file 13054_2021_3885_MOESM3_ESM.jpg]

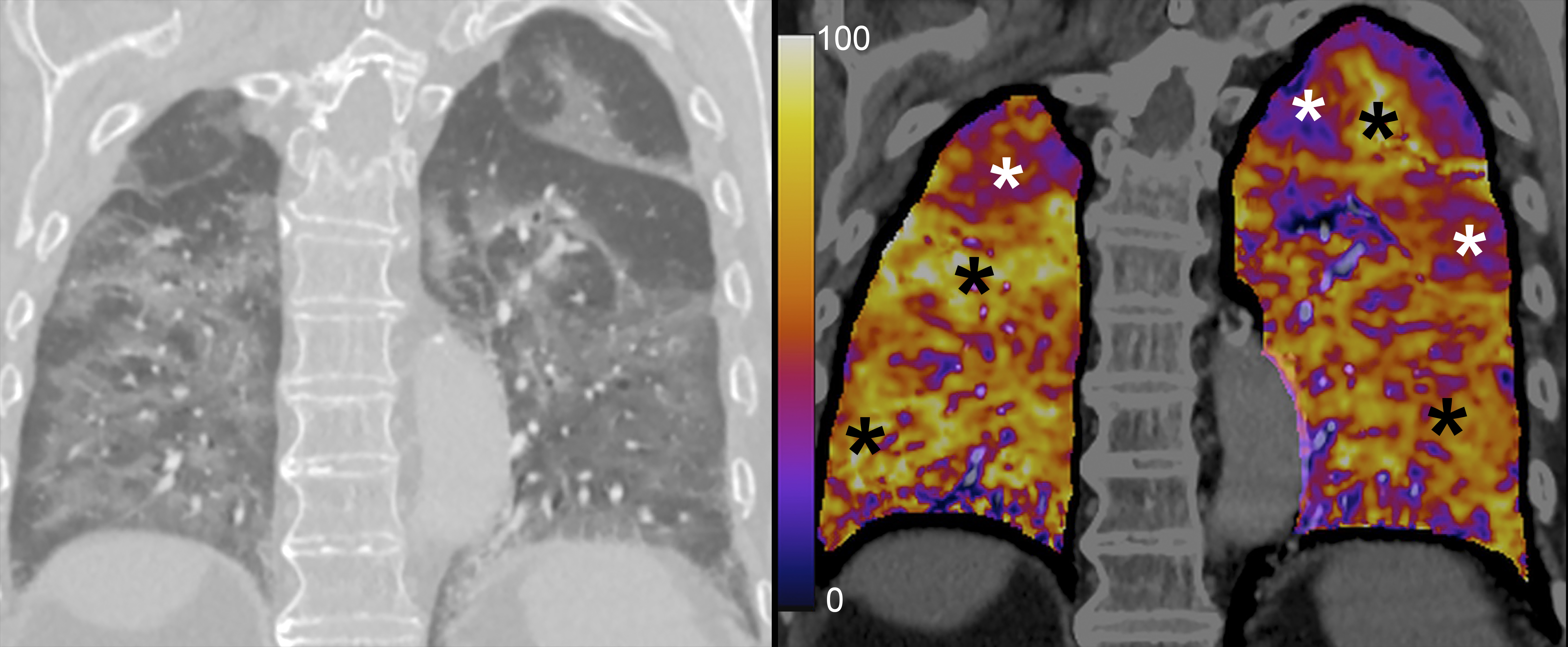

Supplement: Supplementary file 4 — Additional file 4: Figure S2. Conventional and color map sCTA coronal images. Predominance of hyperperfused areas of airspace disease over hypoperfused healthy parenchyma. Excluded from randomization. 73-year-old female patient. RT-PCR confirmed COVID-19, 7 days since symptom onset. PaO2/FiO2 ratio was 133. She was admitted to the ICU, managed with IMV. She was in the hospital 21 days until discharge. CT severity score: 13; sCTA perfusion score: 10. Extensive lung air space disease, in which there is diffusely increased blood perfusion toward areas with airspace opacification. There is an extension predominance of hyperperfused areas (black asterisks) of airspace disease over hypoperfused (white asterisks) in apparently healthy parenchyma. [file 13054_2021_3885_MOESM4_ESM.jpg]
