# Supplementary material for: Sildenafil for treating patients with COVID-19 and perfusion mismatch: a pilot randomized trial
Source: Crit Care. 2022 Jan 3;26:1. doi: 10.1186/s13054-021-03885-y (PMC8721481; doi:10.1186/s13054-021-03885-y)
Supplement: Supplementary file 5 — Additional file 5: Table S2. Estimated effective fractional concentration of oxygen in inspired gas of low-flow and reservoir oxygen delivery devices. [file 13054_2021_3885_MOESM5_ESM.docx]

**Additional file 5: Table S2. Estimated Effective Fractional Concentration of Oxygen in Inspired Gas of Low-Flow and Reservoir Oxygen Delivery Devices.**

| Device | Flow (L/min) | FiO_2_ (%) |
| --- | --- | --- |
| Simple nasal cannula | 1  2  3  4  5 | 24  28  32  36  40 |
| Reservoir mask | 15 | 80 |
| Venturi Mask | 3  6  9  12  15 | 24  28  35  40  60 |
| High-flow nasal cannula | 40-60 | 30-100 |
